# Community-led landscape regeneration: A review of and framework for engagement in restoration initiatives

**DOI:** 10.1007/s13280-025-02236-3

**Published:** 2025-09-20

**Authors:** David Brown, Jennifer Gabrys

**Affiliations:** Department of Sociology, University of Cambridge, Free School Lane, Cambridge, CB2 3RF UK

**Keywords:** Communities, Community engagement, Landscape regeneration, Natural regeneration, Restoration

## Abstract

With growing calls for people-centred and equitable approaches to regeneration and restoration, this review paper contributes to enhancing understanding of the role of communities in restoring landscapes across the world. Addressing the lack of clarity around tangible pathways for equitable and inclusive forms of landscape regeneration, we focus on exploring the practices and forms through which communities engage with landscape regeneration and restoration. We undertake a systematic review of an international selection of community-based landscape regeneration initiatives worldwide to better understand how communities engage with, manage and lead regeneration practices. We map landscape regeneration and restoration initiatives across international contexts based on four themes around community organisation, land ownership, engagement and land values. Borne out of this review, we propose an analytical framework for community-based landscape regeneration in order to support and mobilise more democratic and socially just approaches to ecological regeneration initiatives.

## Introduction

Large-scale landscape restoration and regeneration initiatives have emerged as a central strategy for responding to climate and biodiversity crises worldwide. The UN’s Kunming-Montreal Global Biodiversity Framework is working towards a target of effectively restoring at least 30 per cent of the world’s degraded ecosystems by 2030 (CBD 2022). However, such large-scale and wide-ranging sustainability transitions could negatively impact marginalised rural land users and clash with social equity goals (Hegwood et al. 2022). The long-term success of regeneration initiatives requires integrating social and ecological goals to avoid entrenching inequalities within rural populations (Lofqvist et al. 2023).

There are growing calls for restoration and regeneration programmes to develop more equitable approaches to restoration by centring the needs and perspectives of communities throughout the design, implementation and management of landscape initiatives (Erbaugh et al. 2020; Osborne et al. 2021; Maniraho et al. 2023) and to address multi-dimensional justice concerns (Stanley et al. 2025). Equitable solutions to ecological protection and recovery are dependent on complex landscape governance arrangements and interactions, which raise wider questions around multi-scalar power relations and land control in rural areas (Weng 2015; Lofqvist et al. 2023) and around the integration of diverse communities into landscape-scale visions of ecological restoration (Chazdon et al. 2016; Urzedo et al. 2022a). However, the pathways of action for realising equitable solutions remain highly contested and unclear. As Lofqvist et al. (2023: 135) comment, there is a need to “close the gap between the increasingly recognised imperative of making restoration people centred and participatory and the way restoration science and policy are conducted in practice”.

In this paper, we focus on exploring the practices and forms through which communities engage with landscape regeneration and restoration. Through undertaking a systematic review of community-led landscape regeneration initiatives worldwide, we advance knowledge on the nature and scope of community-oriented landscape regeneration practices. We specifically ask: Who is the “community” in regeneration projects? How do land ownership patterns influence community connections with regeneration initiatives? How do communities engage in restoration and regeneration programmes, whether through initiating, participating in or sustaining them? What values do community-landscape regeneration projects express, whether through attention to environmental change, cultural heritage, livelihood or other factors? In exploring these questions, we contribute to synthesising and advancing scholarship on regeneration and restoration social science, which relative to conservation social science is less well established.

Alongside the moral imperative to strengthen community rights and benefits, there is increasingly wide recognition of the important role that communities play in the stewardship of landscapes and socio-ecological systems around the world. A substantial base of social science research on conservation has demonstrated the ongoing, active role of Indigenous Peoples and Local Communities (IPLCs) in effective environmental protection. These studies generally show that equitable and participatory approaches to environmental governance are linked to positive environmental and social outcomes (Oldekop et al. 2016; Dawson et al. 2021; Huber et al. 2023), including on Indigenous lands and self-determined territories (Sze et al. 2022; M’Sit No’kmaq et al. 2021). More equitable forms of environmental governance include recognition of rights and contributions of IPLCs, integration of diverse values, knowledge forms and cultural practices and community inclusion in decision-making and control of interventions (Dawson et al. 2024a).

Community-based management of environmental interventions is more locally appropriate and draws on traditional ecological knowledge systems and customary practices (Pascual et al. 2021) and can facilitate enhanced community well-being and cultural resilience (Dawson et al. 2021). Moreover, greater local legitimacy is brought to environmental interventions through community leadership and enhanced participation in decision-making and monitoring (Sze et al. 2022; Huber et al. 2023). As a result, scholars have emphasised the importance of more locally appropriate site-level management, devolution of power to the local level and fair and inclusive processes.

Yet evidence of correlation between community involvement and effectiveness of outcomes is inconclusive and highly contextual, shaped by the complexities and qualities of governance arrangements (Zhang et al. 2023). Community-based forms of environmental governance can be exclusionary and lead to unequal outcomes (e.g. sharing of benefits), underpinned by the often-limited scope and definition of the “community” (Ribot et al., 2010; Baruah 2017). Compared to conserving existing ecosystems, the role of communities in regeneration and restoration is less well understood, even as they offer distinct opportunities for community engagement through designing landscape characteristics (Weng 2015).

Our research particularly considers initiatives that are community-led to evaluate how these projects progress within wider regeneration and restoration contexts that are often led by conservation NGOs, large landowners and expert science, which typically have adopted a more technocratic approach to regeneration and restoration (Mehta et al. 2019; Maniraho et al. 2023; Stanley 2024). This might include landscapes with long-standing connections to place or in autonomous Indigenous territories (Coombes 2007; Latulippe and Klenk 2020), a phenomenon that has been less well researched compared to community participation in externally controlled projects.

This paper contributes to a growing body of social science literature and longer-standing political ecology scholarship which adopts a critical approach to researching the role of communities in addressing environmental and sustainability challenges. This scholarship challenges—and moves beyond—typical visions of communities as monolithic, harmonious entities with clearly defined borders, instead understanding these as multi-dimensional, internally complex and dynamic sites of contestation across Global North (Walker 2011; Markantoni and Woolvin 2015; Bower et al. 2024) and Global South contexts (Agrawal and Gibson 1999; Belsky 1999). It also identifies the complex ways that community-based action is intertwined with and shaped by state agendas (Creamer 2015; Taylor Aiken et al. 2017; Sharma et al. 2023).

While community-oriented research has primarily focussed on climate and conservation governance, there is scope for a critical approach to researching communities to be extended to restoration and regeneration settings where communities tend to be similarly defined in dominant policy discourses and practices in limited ways. Emerging social science research on regeneration and restoration case studies, especially in Europe, is focussed on exploring land conflicts and divergent values that form around regeneration and restoration interventions within rural populations (Vasile 2018; Murphy et al. 2022) and the challenges of equitable governance (Martin et al. 2023). Scoping reviews have also developed evidence-based frameworks for social-ecological and community-based restoration (Fox and Cundill 2018; Maniraho et al. 2023). In their review of community engagement in ecological restoration, Fox and Cundill (2018) identify several barriers to the sustainability of community-based approaches, ranging from power inequalities to assumptions about “communities”.

In this paper, we review and map different landscape regeneration projects and programmes across the world according to a four-part thematic framework. These themes consider: (1) how communities are designated, (2) how property relations can influence regeneration initiatives, (3) whom or what engagement practices involve and mobilise and (4) how land values can guide projects across their inception, implementation and long-term management. This analytical framework contributes to advancing knowledge of the spectrum of community-based landscape regeneration projects and how these are governed, developed, practised and implemented across international contexts. Based on this review, we conclude with a set of recommendations for advancing and enabling community-oriented restoration and regeneration projects.

## Methods

This study evaluates a range of community-based ecological restoration and regeneration initiatives through a systematic literature review. We undertook keyword searches (see Table 1) using academic literature databases (Scopus, Web of Science, Google scholar), grey literature (e.g. environmental NGOs, community organisations) and web search engines. The search involved scanning English-language peer-reviewed literature and web resources to identify initiatives focussed on landscape regeneration, restoration, rewilding or recovery which engaged to some extent with communities.

**Table 1:** The search terms were constructed by combining 12 keywords relating to landscape regeneration and restoration themes (column 1) and 3 keywords relating to community involvement themes (column 2). Using the Boolean operator "AND", each of the keywords in column A were searched for in combination with each of the column B keywords

1. “Landscape restoration” OR “Landscape regeneration” OR “Landscape recovery”	AND	1. community-based OR community-oriented OR community-led 2. “community control” OR “community management” OR “community governance” OR “community involvement” 3. Co-create* OR co-develop*
2. “Ecological restoration” OR “ecological regeneration” OR “ecological recovery”		
3. Rewild*		
4. “Forest restoration” OR “Forest and landscape restoration” OR “forest regeneration” OR “forest recovery”		
5. “Woodland restoration” OR “woodland regeneration” OR “woodland recovery”		
6. “Peatland restoration” OR “peatland regeneration” OR “peatland recovery”		
7. “Mangrove restoration” OR “mangrove regeneration” OR “mangrove recovery”		
8. “Wetland restoration” OR “wetland regeneration” OR “wetland recovery”		
9. “Marine restoration” OR “marine regeneration” OR “marine recovery”		
10. “Tree planting” OR Reforestation		
11. “community forest*” OR “community woodland*”		
12. Farmer-managed natural regeneration		

From the initial search, we screened academic articles in two steps: abstract and keywords screening, followed by full article screening. Screening was undertaken based on two criteria. First, there must be a focus on agendas seeking to restore, regenerate or recover landscapes, meaning that programmes that solely had conservation goals were removed. Second, the selected initiatives had to indicate some level of community involvement with regeneration and restoration *practices*, demonstrating substantive roles for communities in the design, delivery or implementation of restoration and regeneration programmes. Importantly, these roles should go beyond (although not necessarily exclude) involvement in consultative processes or surveys. Articles were thus included in the review if they met both of these criteria.

Data were extracted from each paper in order to examine different approaches to landscape regeneration and restoration, covering the following aspects: type of restoration; forms of community engagement and leadership; governance characteristics; funding flows; environmental policy and target orientation; scope of communities; landownership contexts; inclusion of local knowledge and participatory knowledge-building. These data points were synthesised into a four-part analytical framework that evaluates community organisation, land ownership, engagement (including initiating, participating in and sustaining projects) and land values. The four overarching themes emerged from the data as being critical to explorations of questions around communities and landscape regeneration.

## Results

The final selection of papers (*n* = 81) in this review included a plurality of community-based regeneration and restoration initiatives, spanning locations across the world, including the UK, the Philippines, Ghana, the US, Mexico and multiple other locations. Within the scholarship, there was a predominant focus by researchers on restoration initiatives in the Global South, reflecting a bias identified in the conservation literature base (Dawson et al. 2024b). Further cases of community-based regeneration and restoration were then accessed through searching grey literature and web resources.

The initiatives identified in the review vary in their scope and approach, ranging from those focussed on a narrower set of discrete goals (e.g. tree-planting) to projects aiming to recover habitats and ecosystems more holistically, including at landscape scales. These also incorporate a range of ways in which communities engage with landscape restoration and regeneration practices. The majority of cases in this review draw on the terminology of restoration, the prevailing term used within academic literature and policy spheres to describe “the process of assisting the recovery of an ecosystem that has been degraded, damaged or destroyed” (Gann et al. 2019, p. S7). However, initiatives draw from a range of associated paradigms and concepts, including rewilding, recovery, reclamation, rehabilitation and regeneration. While ostensibly addressing common goals of driving ecosystem and landscape recovery, each of these indicates a plurality of objectives, practices, scales, motivations and visions, with varied implications for the role of communities (Chazdon et al. 2016; Gabrys et al. 2022; Mutillod et al. 2024). We use the self-described terms of the study, project or community when referring to the different types of initiatives.

### Community-led landscape regeneration: A review and framework

In the following sections, we discuss the four main themes that emerged in the literature review, as follows: (1) Community organisation: Who is the “community” in regeneration projects? (2) Land ownership: How does land ownership and control influence community-based regeneration practices? (3) Community engagement: How do communities engage in restoration and regeneration programmes, whether through initiating, participating in or sustaining them? (4) Land values: What values do community-landscape regeneration projects express, whether through attention to environmental change, cultural heritage, livelihood or other factors? These themes are outlined in the table below (Table 2), highlighting the organisational characteristics, key trends identified in the literature review and relevant actors.

**Table 2:** Community-based regeneration framework

Themes	Organisational characteristics	Key trends	Actors
1. Community organisation	Community-based organisations (CBOs)	Critical involvement of CBOs in the design and implementation of restoration initiatives, demonstrating a diverse set of goals and agendas	Members of community-based organisations
	Decentralised natural resource governance	CBOs tending to form around direct natural resource and land users, tied to wider process of decentralisation of natural resource governance	Community-based organisations; direct land users and natural resource users
	Wider community engagement	Some initiatives hold aims to engage with wider sections of the communities, e.g. training, education, benefit-sharing, yet these are often limited in practice Questions over the representation of the wider community in restoration initiatives	Community-based organisations
	Risks of marginalisation and elite capture	Some evidence of elite capture of community-based regeneration and of some groups being excluded from benefits Few focussed efforts on involving marginalised groups	Community leaders
2. Land ownership	Community participation on state-owned lands	Majority of initiatives take place on state-owned lands, with community involvement typically limited in extent	State bodies, municipalities, international organisations, NGOs
	Community management of land	Prominence of community-managed lands and resources based on negotiated agreements between communities and external actors Evidence of lack of livelihoods improvement, limited democratic accountability and community empowerment	Community-based organisations, Municipal/local institutions, NGOs
	Community ownership of land	Small number of community-owned lands (e.g. community forests), showing greater community control over the nature and scope of the benefits of restoration initiatives, yet subject to wider barriers and pressures	Community-based organisations, Indigenous communities
	Land rights and access	Community-based initiatives sometimes driven by wider, ongoing struggles for land rights, access, Indigenous territorial claims	Community-based organisations, Indigenous communities
3. Community engagement: Initiating, Participating, Sustaining	Community involvement in initiating restoration projects	Few cases of community leadership, more commonly cases involve lower levels of control over the design and development of the project and a greater extent of collaboration with other actors, or multi-stakeholder arrangements	Communities, international NGOs, international organisations, state bodies
	Community participation in external restoration programmes	Spectrum of community involvement in external restoration programmes, ranging from minimal participation as implementation or monitoring partners to community organising and collaboration on project design	Community-based organisations, volunteers, citizen scientists
	Community involvement in sustaining restoration projects	Communities in primary control of restoration projects yet with limited resources to sustain these, reliance on external funding and support Importance of network building for sharing of resources, knowledge across community-based regeneration	Farming associations, cooperatives, community-based organisations
4. Land values	Market-based interventions	Limited to no identified examples of market-based interventions working in favour of communities	Financiers, corporations, start-ups, communities
	Links to land-based livelihoods	Many community-based regeneration initiatives are tied to land-based livelihoods, either directly (e.g. integrated with restoration practices) or indirectly (e.g. financially supporting the restoration activities)	Community-based organisation, natural resource users, farmers
	Integration of community-based knowledge and values into regeneration initiatives	Limited inclusion of local knowledge and values in the majority of cases, with continued domination of Western scientific knowledge systems Local knowledge and values integration exists on a spectrum, bound up with community control and land sovereignty, ranging from participatory knowledge-building to species and site selections to incorporation of traditional or Indigenous knowledge into plans and to participatory planning and co-design	Communities; Indigenous communities

#### Community organisation: Who is the “community” in regeneration projects?

It is important to move beyond simplistic representations of communities and to adopt a critical viewpoint on who is and is not included as part of community-landscape restoration and regeneration initiatives. Communities are characterised by heterogeneity, inequalities and internal power imbalances, structuring how the benefits of restoration programmes are distributed and who holds decision-making capabilities around these (Elias et al. 2022). Environmental initiatives are often supported and driven by privileged sections of the community, resulting in the marginalisation and exclusion of others (Kenis and Mathijs 2014; Fox and Cundill 2018).

Our review found that the majority of regeneration initiatives (81%) are broadly oriented around “communities of place”, in other words, those residing in close proximity to regeneration sites as participants, volunteers or employees in the design and/or delivery of restoration programmes and practices. These interventions are embedded in particular places defined by geographic boundaries. The exceptions to this are citizen science and volunteer projects characterised by “communities of interest” forming around shared socio-ecological goals, which for instance include university students from the wider region or online participants.

Grounded in geographic communities, this review highlights the critical involvement of community-based organisations (CBOs) in the design and implementation of restoration initiatives (demonstrated in 43% of papers), including by connecting and establishing formal institutions and associations, groups, cooperatives and networks. While they may have agreements or forms of collaboration with other bodies, CBOs are grassroots, membership-based entities that generally operate independently of the government. They tend to be comprised of direct land users or community members with interests in natural resource management. While CBOs may have aspirations and targets to reach those in the wider community and to pursue cooperative goals and activities centred around community development (Shaban et al. 2016), the scope of community in such contexts is typically defined by institutional membership. In some cases, financial benefits from the proceeds of restoration projects (e.g. carbon sales) and land economies developed by CBOs are shared with the wider communities (Blay et al. 2008).

In relation to forest governance, Fischer et al. (2023: 1345) highlight the importance of formal CBOs, which they define as those holding “legal recognition of local management authority by the state”. By decentralising resource management authority to the community level, there is the potential for restoration initiatives to be carried out in a more locally responsive way, to connect to rural livelihoods and to more effectively draw from place-based socio-ecological knowledge. Fischer et al. (2023: 1345) argue for the “need to move beyond structured stakeholder consultations and project-based participatory forums to build more durable and empowered local institutions that can enable socio-ecological benefits over the long term”. CBOs can offer community-led decision-making based on an organisational structure (Kamelamela et al. 2022). However, questions remain over the extent to which CBO management is representative of the wider community or includes a plurality of interests, values and perspectives.

In this review, we found literature that documents the diversity of CBOs in their scope, objectives and membership. Nevertheless, the majority (74%) of CBOs consisted of pre-existing groups that then connected to restoration efforts, either by collaborating with other actors or leading initiatives themselves. These projects include CBOs organised around the sustainable management of various natural resources, for instance forests or water, where CBO membership is often comprised of a particular set of land users and those with some form of land-based or technical expertise. These groups oriented around natural resources are likely to capture the main benefits of the initiatives, for instance through payments for participation or through boosting livelihoods.

A section of the CBOs (22%) identified in this review are based on organising farming communities, for instance farming associations and cooperatives. In Western Kenya, Peterson et al. (2018) detail an environmental CBO, Nyando Development Community Centre for Environmental Conservation, which adopted a community-based model to combat soil erosion in the area through mobilising the formation of “gully rehabilitation trusts” across five areas, comprised of farmers directly affected by soil erosion. They aim to build capacities of trust members to address environmental degradation by organising training, including to monitor and evaluate restoration activities and for financial management.

The review highlights farmer associations in West Africa organised around farmer-managed natural regeneration (FMNR) practices to address land degradation and deforestation. One example of this is in the Talensi District of Northeastern Ghana (Kandel et al. 2022). The project facilitated the formation of community groups which involved training on FMNR practices. The “lead farmers” from this group then played a key role in knowledge-sharing and in mobilising the adoption of regenerative practices by the wider community (Weston et al. 2015).

Elsewhere, some CBOs (31%) involved in restoration and regeneration are comprised of a coalition of different natural resource users and sections of the community. For example, in the USA, a CBO coordinating the restoration of the Colorado River delta is made up of different sections of the communities, including farmers, fisherfolk, hunters, aquaculturists, as well as members of the Cucapá tribe (Hinojosa-Huerta et al. 2005).

A sizable proportion of CBOs (34%) in the review aim to incorporate groups and sections of the community beyond natural resources. For instance, Olukoye and Kinyamario (2009) highlight a case in Kenya where community-led initiatives addressing desertification through tree-planting activities are organised by a diverse range of groups, including a Catholic religious group and women’s group. More often, however, CBOs continue to be primarily organised by natural resource and land users while involving efforts to bring in the wider community, including residents in the area, to engage with restoration activities, for instance through training and education or the sharing of benefits. In Uganda, a number of CBOs have emerged as stakeholders in the governance of Mount Elgon national park, undertaking restorative activities (Shaban et al. 2016). The CBOs train members in developing practical skills for restorative activities—notably with techniques for tree and grass planting—yet also mobilise them to run community tree nurseries, to engage with collaborative forest management implementation and to carry out marketing activities (e.g. for non-timber forest products).

However, despite the participatory framings of many community-based approaches, there tends to be limited inclusion of the wider community. The majority of CBOs engaging in restoration are being driven by particular sections of the community, often forming around landownership and natural resource control. Alongside the uneven power dynamics between communities and external institutions, the review also draws attention to how community-based approaches can result in marginalisation and elite capture. Restoration initiatives take place along contours of established and emerging unequal within-community power relations, for instance primarily benefitting large landowners and other powerful actors, with others becoming excluded.

In Southwestern Ghana, Baruah (2017) highlights how a CREMA (Community Resource Management Areas) involved in supporting tree-planting on farmland led to elite formation and capture. It is argued that through the work and funding of state bodies, international NGOs and multilateral organisations, CREMA activities in the area have exacerbated inequalities by reinforcing the powers of existing elites and facilitated the emergence of a new elite, while also promoting the privatisation of tree tenure on farms and demonstrating a lack of democratic accountability to local populations. Elsewhere, in Northeastern Ghana, Kandel et al. (2021) detail how the complex hierarchies of land tenure and natural resource control mediate the unequal distribution of benefits among farmers from natural regeneration initiatives. These examples raise questions of how community cohesion and land-based conflicts can mediate the scope of communities in pursuing restoration goals.

Despite the risks of marginalisation, only two cases that we identified in our review explicitly included aim to involve marginalised groups. In the USA, Andrews et al. (2022) describe a two-year community science programme undertaken as part of the university-led Duwamish Floating Wetlands Project. This initiative adopts an environmental justice approach by engaging with marginalised sections of the communities living near the wetlands. A handful of community-based initiatives are also initiated by and include Indigenous groups, or seek to incorporate Indigenous-led decision-making into collaborative organisational structures with other partners (Fox et al. 2017; Kamelamela et al. 2022). Engagement with marginalised groups within restoration and regeneration initiatives was thus an area for further development, since these communities are more likely to be left out of decision-making processes that directly affect their livelihoods.

#### Land ownership: How does land ownership and control influence community-based regeneration practices?

Land ownership and control are an important factor in determining how or whether community-based landscape regeneration can take place. Efforts to restore and regenerate the landscape cannot be separated from recognition of land rights and of land sovereignty, or from historical and ongoing land dispossession (Barra and Jessee 2024). There are fears that restoration initiatives could exacerbate rural and land inequalities and further marginalise communities, amid unequal power structures and processes of “green grabbing” (Fairhead et al. 2012), where large and wealthy rural landowners can disproportionately benefit from investments in restoration and regeneration (McIntosh et al. 2023).

In this review, we found that the state or the municipality is the primary landowner in around two-thirds (62%) of restoration and regeneration initiatives identified. On these lands, regeneration projects are primarily led by external stakeholders, including state bodies, but also international organisations and NGOs working under partnership models, with community involvement typically being limited in extent. In these circumstances, communities have no hold over the land themselves and tend to be participating actors in land-based projects led by others, for instance as volunteers or citizen scientists.

Distinct from this model, we identified a small number of cases in which regeneration activities are being carried out on community-owned land (16%), often community forests. One notable example is in the Intag Valley of Ecuador where a local NGO *Defensa y Conservacion Ecologica de Intag* purchased land and titled it to communities in order to establish communal reserves and reforestation projects, alongside on-farm tree-planting (Wilson and Rhemtulla 2016). Facilitated by international donor funds, the NGO also provided training and financial support (e.g. for materials) for community members to propagate and plant native tree species. While the NGO initiated the projects, these were formed in response to community concerns around the impact of deforestation on farming livelihoods. A key goal of the project, as Wilson and Coomes (2019: 156) outline, was to create “strongholds in communities—areas of land owned communally that could not be sold […] without a community decision-making process”. In this case, there was a high level of community action where, for instance, residents maintained planted trees through communal work parties.

In the community forests of Mexico, which are designated as land held under common property, community organisation and autonomy intersect with forest recovery (Hernandez-Aguilar et al. 2021). In the Mixteca Alta region in Oaxaca, legal and institutional frameworks are identified to underpin community governance as key in facilitating reforestation practices. Notably, the community assemblies established new rules for sustainable forest use, including regulation of grazing practices, and organised regular reforestation events which drew on the long-running practices of *tequios,* where members are obliged to contribute labour towards community needs (Hernandez-Aguilar et al. 2021). The authors highlight the endogenous factors within the community forests that facilitated the significant enhancement of forest cover in the area, notably self-organisation capacity, local leadership and social learnings.

The case of the Khasi Hills initiative in India similarly highlights the importance of community forests as local organisational structures that provide the social foundations for forest restoration (Poffenberger 2015). In response to growing threats to their forest ecosystems, 62 villages across 10 Indigenous governments within the Umiam Valley formed together to establish the Khasi federation as a way of coordinating the restoration of their community forests. The restoration practices were then organised at the local level through working committees. As the Khasi communities owned the land, they were able to quickly move to organise themselves through local institutions to work towards forest restoration goals without the need for approval from the state or other landowners. While an initial income stream was facilitated by a collaborating NGO, the intention is for the federation to become self-sufficient in leading on forest restoration plans as part of a 30-year climate adaptation strategy (Poffenberger 2015).

In Scotland, community woodlands and forests have grown substantially in numbers since land reform in 2003 (Sharma et al. 2023; Martin et al. 2023). Community ownership of land in Scotland has been driven by those seeking land justice in a context of long-standing high concentration of landownership and dominance of large estates, as well as efforts to develop local economic resilience and to restore the landscape, particularly peatlands and woodlands. For instance, in Langholm, the community bought out a large estate, previously a grouse moor, and developed multiple, interconnecting goals around the community, nature and climate. Through community ownership of land, the aim is to build nature-based economies in pursuing socio-economic and landscape regeneration, including the management of a community-based nature reserve, involving peatland restoration and wetland creation (The Langholm Initiative 2025).

Through ownership of land and natural resources, communities are able to exercise greater autonomy in relation to the nature and scope of the benefits of restoration initiatives and can more closely determine how restoration plans can be oriented towards community needs and livelihoods. Yet, despite this enhanced sense of control and authority, community lands are also the subject of external influence and pressures, with community-based initiatives substantially shaped by funding structures and support systems (Sharma et al. 2023), along with international and national policy agendas to reverse biodiversity loss and to address climate change.

Beyond community forests, a greater number of cases in this review relate to community-managed lands (29%) where the community do not own the land being recovered yet hold management rights and responsibilities for it. This might involve CBOs or land users leasing land from state bodies or private landowners, as part of either statutory or customary tenure arrangements. Customary tenure is not often legally recognised, and a lack of clear land tenure can contribute to deforestation and land degradation (Mansourian et al. 2014) or otherwise constrain community-based and equitable land stewardship (McElwee 2011; Baruah 2017).

Customary land tenure arrangements are found in cases of FMNR in Sub-Saharan Africa where the primary focus is on restoring farmlands, yet in some cases, initiatives have extended the scope of FMNR beyond participating farms towards restoring degraded woodlands and forests held in common (Weston et al. 2015; Kandel et al. 2021). In the context of an FMNR initiative instigated by the international NGO world Vision in Northeastern Ghana, the development of community FMNR sites—to be governed as “community forests”—was in tension with customary land and tree tenure systems. Resisting a “top-down community forest model”, households continued to maintain certain rights to the land (Kandel et al. 2022).

Since the 1990s, it has become increasingly common for governments across the world to devolve rights and responsibilities for the management of natural resources to local levels, ascribed under various monikers as Participatory Forest Management, Community-based Natural Resource Based Management or Community-Based Forest Management, among others (Kothari et al. 2013; Lund 2015), notably in parts of Sub-Saharan Africa (Baruah 2017) and South-East Asia. Here, agreements are made between communities and the landowners, typically governmental or municipal bodies but not always, to co-manage a piece of land and resources, sometimes mediated by third-party organisations such as international NGOs. Community-based natural resource management agreements are commonly based on a negotiated time period and set of conditions (Mansourian et al. 2014).

In contexts of co-management or community-based management in which communities do not own the land and with unequal power dynamics between communities and external actors at play, it is not always clear how communities can substantially define the nature and scope of regeneration initiatives, along with the benefits emerging from them. For some communities, involvement in restoration initiatives is wrapped up with wider struggles for land rights, access, Indigenous territorial claims and recognition (Blay et al. 2008) and with confronting the legacies of land dispossession (Barra and Jessee 2024). In particular, Elliott et al. (2019) detail how the engagement of the community of Ban Mae Sa Mai in Thailand with reforestation efforts was mainly driven by aims to demonstrate responsible forest stewardship as a way of supporting territorial claims and resolving conflict with the national park authority. Moreover, Mansourian et al. (2014) highlight that a key driver of community engagement in co-management of forest restoration in Madagascar is the potential to strengthen land tenure security and to enhance recognition of land rights through management agreements.

In the Philippines, land tenure for farmers engaging in agroforestry, forest restoration and carbon sequestration initiatives is based on 25-year community-based forest management agreements with the government (Lasco et al. 2010; Gregorio et al. 2020). By collaborating with “People’s Organisations”, the government is promoting community-based forest management as a way of delivering its environmental goals through multi-level governance structures. However, despite the decentralised management structures, it has been identified that communities are granted a highly limited set of decision-making and project design capabilities (Wiset et al. 2023).

More widely, the extent to which decentralisation of land management can strengthen communities’ claims to land and to land tenure is highly contested (Mansourian et al. 2014). Critical scholars have observed that in many cases devolved natural resource governance has been associated with limited democratic accountability and the continued dominance of state priorities (Baruah 2017; Mawutor and Hajjar 2022). In theory, participatory forms of natural resource management are expected to instigate community empowerment and livelihood development along with improved ecological outcomes. However, the wider evidence base has demonstrated mixed outcomes, and improvements for community livelihoods have not materialised (Kothari 2013; Lund 2015).

While these programmes should incorporate communities as equal collaborators in environmental initiatives, conservation social science and political ecology scholars have highlighted the false promises of participatory forms of natural resource-based management, which have been critiqued as perpetuating state-dominated agendas, as leading to elite capture and conflicts, or as not being meaningfully empowering for communities (Cooke and Kothari 2001; Ribot et al. 2010; Lund 2015). While there are substantial barriers to the effective devolvement of governance to the community level, Diver (2016) highlights pathways through which Indigenous communities can stake claims to territory and rights through co-management regimes.

#### Community engagement: How do communities engage in restoration and regeneration programmes, whether through initiating, participating in or sustaining them?

Community engagement in landscape regeneration can take a variety of often overlapping forms and can include varying levels of community representation, influence and control of restoration agendas. This section identifies three key areas of community involvement, including the initiation and leadership of projects, different forms of community participation that are typically led by state or other actors, and community-based approaches to sustaining projects. The division into these three areas is influenced by the wide-ranging understandings of community engagement and the spectrum of forms of community participation covered by sustainability initiatives, as captured by the conservation literature (Zhang et al. 2023; Dawson et al. 2024a), while also highlighting the importance of both how restoration projects get off the ground and what is needed to keep projects going for the longer term.

##### Initiating

This review highlights a range of community-led and initiated approaches to restoration (33% of cases). Cases of community leadership in restoration can incorporate community forests where the community has territorial autonomy and significant levels of authority and control over land management and decision-making. Such arrangements are almost entirely locally led, with only some support (material, technical) from external actors in delivering the restoration programme. It can be understood that these refer to situations in which communities are in primary control of restoration projects or “centred as the key agents” (Armitage et al. 2020: 3) of restoration and regeneration actions.

More commonly, cases involve lower levels of control over the design and development of the project and a greater extent of collaboration with other actors. The review demonstrates that communities co-lead restoration initiatives as part of collaborative efforts in which they have some leadership and decision-making responsibilities (26% of cases). Communities may work as partners in a collaborative manner with other stakeholders in developing and implementing restoration and regeneration projects, particularly state bodies and NGOs. NGOs sometimes play a bridging role between the communities and the state. The category of co-leading refers to a wide range of governance arrangements and actors and varying levels of community control in practice. Alternatively, community-based projects can refer to multi-stakeholder set-ups (11% of cases) in which community voices are less influential (e.g. Derak et al. 2018).

Mustonen et al. (2022) detail two examples of collaborative efforts to manage peatland and river restoration projects in Finland, as part of the “landscape rewilding programme”, where community associations and Indigenous groups co-lead initiatives with state and municipal environmental bodies. With cases of FMNR in Sahel regions, the idea is that NGOs and environmental institutions initially encourage and materially support the adoption of FMNR in rural areas, with the expectation that farmers will subsequently take ownership of FMNR practices.

Supporting research findings in the conservation sphere (Calfucura 2018; Dawson et al. 2024a), many restoration projects in this review are labelled as “community-based”, “community-driven” or even as “community-led”, yet the role played by communities is often limited to organisation and implementation of the projects. The fundamental decision-making and defining of the restoration targets and goals are set externally by the state, NGOs or international bodies, indicating a misrepresentation of the role of communities in these restoration projects. These indicate identified processes of “community-washing” of environmental projects (Ptak et al. 2018) and of the term “community” being instrumentalised in Western, neoliberal contexts as a way of pursuing and delivering policy goals around the environment and climate through voluntary labour (cf. Taylor Aiken et al. 2017).

##### Participating

A significant proportion (41%) of community-based restoration initiatives are directly and tangibly driven by wider programmes to restore the land, led by the state, local or international NGOs or international organisations (e.g. the World Bank). National policy objectives tend to be the main driver of restoration projects, yet these overlap with and are influenced by wider international agreements, funds and agendas around landscape restoration and biodiversity. Across these restoration programmes, there is a spectrum of community participation and involvement, ranging from minimal participation as implementation or monitoring partners to a collaborative role with other stakeholders (state, private and/or NGO), supporting findings from the wider literature on programmes covering conservation, natural resource governance and climate-based relocation (McAdam and Ferris 2015; Zhang et al. 2023; Dawson et al. 2024a).

Communities are sometimes recruited by governments as volunteers or workers to carry out the on-the-ground implementation of restoration goals and plans set by the state. For example, in the KwaZulu-Natal province of South Africa, a public programme to restore water catchments involved employing people to eradicate invasive plant species along river streams (Pasgaard et al. 2023). Connecting to both poverty alleviation and ecological restoration policy goals, members of low-income communities are being trained as “restoration workers”. A number of NGO projects demonstrated similar dynamics with community involvement. Tidball et al. (2018) describe a case in which an NGO in the USA led a citizen tree-planting initiative following the Katrina disaster. This involved interested residents undergoing a training programme before planting trees across neighbourhoods of New Orleans, helping to restore the post-disaster urban landscape.

Other cases demonstrate some level of project management responsibilities being devolved to the local level, primarily relating to organisational aspects of restoration projects. This might include community-based efforts to bring together people to become involved in the implementation of restoration plans. Ewane (2024) highlights a state-led forest landscape restoration project in Cameroon’s Western Highlands which is implemented by a community-based organisation in collaboration with an international NGO, working together as organising partners. The partners focus on developing programmes to incentivise land users to adopt forest restoration practices, notably encouraging farmers to plant and manage trees on their lands and in community land reserves.

Moreover, the review highlights small-scale restoration initiatives involving participatory monitoring. In projects led by environmental organisations, start-ups or university researchers, citizen scientists are recruited to engage in monitoring in order to support restoration knowledge-building and project development. In New York state, an academic research project developed a citizen-science protocol for communities to measure the effect of deer browsing on the growth of seedlings (Curtis et al. 2021). Volunteers were trained to use the protocol and to enter in-field data online.

While communities operationally take part in monitoring activities, external actors and institutions determine the parameters of monitoring operations and what these will be used for. Weng (2015) notes that while citizen scientists are increasingly used to assist with data collection and monitoring in conservation and restoration initiatives, these projects tend to be formulated and delivered in a top-down manner, led by scientists and professionals, rather than as a form of knowledge exchange. It was found in their study that even within participatory restoration programmes, unequal power dynamics between professionals and volunteers persists within an organisational expert-lay hierarchy. Yet while this may indicate seemingly minimal forms of community engagement in restoration initiatives, Turnhout et al. (2010) notes that volunteers may feel a sense of empowerment that is not captured by a narrow, incremental definition of participation.

##### Sustaining

Even with those cases in which communities are leading restoration approaches, there are important questions around sustaining these in the long term, including how communities may self-generate funds rather than be reliant on external funding. The review highlights community-led projects which draw from funding and technical support, including from international NGOs, international institutions and state bodies. In these cases, communities are in primary control of restoration decision-making, yet they are reliant on external sources of finance to sustain the restoration activities and to deliver on restoration plans.

In the case of the Nyando Development Community Centre for Environmental Conservation in Western Kenya (Peterson et al. 2018), a key barrier to building the capacity of the “gully rehabilitation trusts” was identified to be the challenge involved with securing continued funding. Seeking to mitigate the short-term barriers of funding cycles, particularly those connected to international initiatives, the NGO has focussed efforts on “creating lasting relationships with farming households” (Peterson et al. 2018: 76). The NGO has also adopted a more creative approach to capacity-building, notably extending their networks to include a wider range of partnerships. In particular, they have forged a partnership with the University of New England to work on co-developing the use of participatory GIS into the NGO’s work and on training community members to use mapping and monitoring techniques to support the gully restoration initiative.

In general, the literature has noted the importance of financial incentives to facilitate continued and inclusive participation by community members in restoration programmes, notably disadvantaged sections of the communities (Denham 2017). Communities may have strong leadership and high levels of control, for instance in case of community-owned land, yet with limited resources to operationalise their restoration goals due to an unfavourable policy climate (Bower et al. 2024) while communities also have unequal capacities to draw from funding opportunities (Sharma et al. 2023). Wilson and Coomes (2019) highlight different dynamics in the Intag Valley of Ecuador where communities leading forest restoration projects voted to either receive income from international donor funds or to collectively volunteer labour. In those communities drawing from a voluntary labour pool, there were higher participation rates in the restoration projects, where the residents were motivated by factors other than financial benefits. Indeed, it has been argued that community engagement based on a financial incentive model in environmental initiatives tends to be short-lived and precarious, dependent on continued external funding (Agrawal et al. 2015).

At the same time, our review identified the importance of network building among CBOs (15%) in supporting community-based landscape regeneration practices. Primarily, network building is practised through the sharing of resources and knowledge on restoration and regeneration, one approach through which CBOs can overcome unequal power relations with external actors to achieve positive outcomes. This tends to involve training or dissemination of practices by a CBO to other relevant CBOs in the region, including techniques for ecological restoration, monitoring and evaluation, alongside understanding the social dimensions of restoration.

For instance, an Irish national network, “Community Wetlands Forum”, has developed to provide support to community organisations involved in peatland and wetland restoration (Flood et al. 2022). CBOs within the network share resources and knowledge with each other, notably having co-developed a training framework, which includes community organising strategies. Another form of cross-community coordination and resource sharing has come in the form of community seed networks in Brazil, where an Indigenous community-led campaign to restore riparian forests fostered the development of the Xingu Seed Network (Sanches et al. 2021; Urzedo et al. 2022b). This initiative includes Indigenous groups, rural producers and family farms, eventually becoming a formal association collectively responsible for the management of native seed collection, supply and sales.

#### Land values: What values do community-landscape regeneration projects express, whether through attention to environmental change, cultural heritage, livelihood or other factors?

It is increasingly widely acknowledged (Lyver et al. 2016; Fox and Cundill 2018; Pascual et al. 2021; Gabrys et al. 2024) that a pluralist approach drawing on diverse values, perspectives and knowledge is vital for the long-term sustainability and equity of restoration interventions. Despite the growing recognition and inclusion of Indigenous and local knowledge systems and the increasing promotion of collaborative governance, many restoration programmes and initiatives are controlled by states, international NGOs or private firms from the Global North. In line with flows of global funding, policy influence and the proliferation of top-down, technocratic systems, Western values and scientific knowledge forms continue to dominate environmental programmes across the world (Latulippe and Klenk 2020; Barra and Jessee 2024). Community-based land values, ways of life, relations to nature and knowledges, including Indigenous sciences and traditional ecological knowledges, have tended to be marginalised or inadequately recognised by externally controlled conservation and restoration projects, indicating cultural and epistemic exclusion (Guibrunet et al. 2021).

In this review, over half of the restoration initiatives (60%) are not explicitly or directly driven by revenue-generating activities or goals. Many of these are tied to state-based restoration programmes and projects or to NGO-led non-profit initiatives which may involve financial incentives to encourage community-based regeneration activities but not ambitions of revenue generation. Of those cases which form to some extent around revenue-generating goals, these relate to a range of channels, notably including carbon markets, biodiversity credits, payment for ecosystems services, alongside energy generation, ecotourism and more sustainable forms of food production and forestry.

These highlight the breadth of restoration and regeneration initiatives covered in this review, reflecting trends in recent decades of the increasing role of market-based and neoliberal approaches to protecting and restoring nature (Apostolopoulou et al. 2021), yet also strong links to land-based livelihoods and land economies. While the sustainability of community-based projects is constrained by access to continued finances, this review was not able to identify examples of market-based interventions which have worked in the favour of communities. Although this review is selective, it is nevertheless indicative of wider documented conflicts between market-based approaches and community benefits. While beyond the scope of this review, it is worth noting that in other areas of environmental governance, there is some limited evidence of positive cases of market-based initiatives, for instance concerning compensating landholders for biodiversity protection and ecosystems services (Ausinheiler 2025). It is vital to consider the ways in which communities could make market-based approaches to restoration work for them.

There are five cases in this review where community-based tree-planting and agroforestry projects are financed by carbon markets (Lasco et al. 2010; Mansourian et al. 2014), ascribing a monetary value to nature in seeking to financially incentivise environmental protection and restoration. For example, Dyer et al. (2014) describe the Nhambita Community Carbon Project in Mozambique which promotes agroforestry. Households receive carbon payments on the basis of area of trees planted, indicating a marketisation of nature recovery that encourages a narrow focus on tree-planting rather than wider landscape regeneration goals. The study highlights limited local control in a project driven by the private sector and the structures of the carbon market where, for instance, the slow development of the voluntary carbon markets led to payments to project participants being delayed.

Elsewhere, community-based regeneration initiatives are tied to financing land economies and to land-based livelihoods (37%), typically through the forestry sector, including the sales of timber (Lasco et al. 2010) and, in some instances, non-timber forest products (Shaban et al. 2016). Restoration approaches based on livelihoods and integrative socio-ecological goals can allay local fears around land-based trade-offs (Fox and Cundill 2018; Fischer et al. 2023). These livelihoods can provide a source of income to financially support the community-based restoration activities which may not have otherwise been viable or appealing to the community.

The review also demonstrates efforts to develop a more sustainable model of forestry to complement the restoration goals, including efforts by NGOs and CBOs to develop sustainable forest certification. For example, in Eastern Tanzania, Loveridge et al. (2023) outline a case in which a community forest is selling timber as sustainably certified, in combination with forest restoration practices. The initiative has multiple goals relating to well-being, forest recovery and equitable governance to be realised through “certified community forests”, where the revenue from the sustainably harvested timber is then used for community developments.

Elsewhere, farming livelihoods are sometimes given a central role in restoration and regeneration programmes (19% of cases), for instance an integrated biodiversity restoration initiative on cattle farms in Malarhagar, Sweden (Stenseke 2009), or an FLR programme incorporating cattle ranchers in Colombia (Calle 2020). With regard to the latter case, ranchers were motivated to participate in the project to address concerns around land degradation and productivity. Yet it was found that these motivations related not only to profit motives but also the farmers’ sense of obligations as stewards of the landscape, their sense of place and intangible values such as healing the land or environmental aesthetics (Calle 2020). With the Langholm Initiative in Scotland, alternative ways of managing and living off the land are envisaged as part of nature-based economies, while also strengthening the community’s connection with land and nature (The Langholm Initiative 2025).

Scholars have called for the empowerment and centring of local, traditional and Indigenous knowledge systems into restoration programmes and projects and a wider decolonisation of environmental knowledge production, as well as recognition of cultural heritage and customary institutions (Mabele et al. 2022; Orlove et al. 2023). In this review, around two-thirds (68%) of restoration initiatives do not indicate inclusion of local knowledge. Across those cases which are being led or co-led by external actors, technical support based on scientific knowledge systems tends to dominate interactions with communities. Of the cases that demonstrate some level of local knowledge and values integration, this exists on a spectrum, bound up with the level of community control in the restoration initiative. As Reyes-Garcia et al. (2019) document, Indigenous and local knowledge has been integrated into restoration programmes and projects in various ways and at various stages, including in planning, implementation and monitoring of restoration activities.

The review highlights the engagement of regeneration and restoration initiatives with participatory knowledge-building and citizen science. These can involve compiling of knowledge on restoration through community-based ecological monitoring (Sullivan and Molles 2016; Helsey et al. 2017), which has become increasingly prevalent in restoration projects and programmes. Community biodiversity inventories involve volunteer groups documenting, often collaboratively with scientists, ecological metrics such as species distribution, abundance and local use. Fabricius and Perreira (2015) highlight the potential for community biodiversity inventories to draw from local and traditional knowledge, and culturally important aspects of local environments. However, these forms of monitoring often only draw to a limited extent from local ecological knowledge and continue to be primarily driven by external scientific goals and frameworks.

Moreover, several cases (17%) demonstrate the involvement of communities in selecting the species and sites that would be most locally appropriate and culturally salient to use in restoration plans based on Indigenous and local knowledge, sometimes referred to as “cultural keystone species” and “cultural keystone places” (Reyes-Garcia et al. 2019). This integration of community knowledge into site and species selection often focuses less on ecological impacts and more on the socio-economic and cultural aspects of regeneration, for instance how the plant species could be used by the communities, or land access and rights (Elliott et al. 2019; Rao et al. 2022).

These projects tend to incorporate goals of knowledge exchange between communities and scientists. For example, a university-led trial restoration project in Thailand sought to invoke two-way knowledge-sharing between villagers and project scientists (Elliott et al. 2019). Communities provided input into decisions on tree plot locations and on traditional uses of different tree species through mapping exercises and walking-based discussions, while university scientists provided training on data collection and reporting.

Ten initiatives identified in this review are more directly and substantially based on traditional or Indigenous knowledge and science, reflecting greater levels of community control and land sovereignty (Coombes 2007; Mustonen et al. 2022). Kamelamela et al. (2022) describe an Indigenous-led restoration initiative, implemented in collaboration with other partners (forestry body, research partners, conservation NGO) within Pu ‘uwa ‘awa ‘a State Forest Reserve in Hawaii, USA. The initiative is based on Native Hawaiian knowledge, practices and ethics, and multi-generational stewardship of forested land. The community also developed a Pu ‘uwa ‘awa ‘a specific geographic information systems “story map” that integrates a wide range of biophysical and biocultural indicators in order to identify the most locally important dimensions of forest restoration (Kamelamela et al. 2022).

Only six cases highlight contexts in which communities are involved closely across the different stages of the restoration process, notably with the planning and design of projects, rather than only at discrete points in projects. Previous research has emphasised the importance of actively involving communities in co-designing restoration initiatives and activities, helping to build collaborative partnerships and address value conflicts (Lyver et al. 2016; Reyes-Garcia et al. 2019). These seek to infuse community-based knowledge into the core aspects of restoration initiatives, including efforts to co-design restoration plans. For instance, in a case of participatory ecological restoration in Morocco, diverse natural resource and land users are involved in identifying restoration priorities, assessing land-use options and setting the restoration plans and procedures, alongside monitoring and evaluation (Derak et al. 2018).

Participatory planning tends to incorporate community inputs into project designs as part of multi-stakeholder collaborative arrangements, including setting agreements on restoration plans. Co-design approaches sometimes form part of experimental research projects led by universities and NGOs to co-develop bottom-up plans for restoration sites, e.g. “living labs” approaches, community mapping (Lupp et al. 2020; Gómez-Ruiz et al. 2022). For instance, a research institute in the USA deployed participatory modelling as part of a co-design approach focussed on restoring wetlands in Louisiana. Communities worked directly with modellers to identify the environmental problems to be addressed, determining priority restoration sites, identifying and reviewing model inputs and evaluating model results based on local ecological knowledge (Hemmerling et al. 2023).

## Conclusion

With growing calls for people-centred, community-based and equitable approaches to regeneration and restoration (Erbaugh et al. 2020; Lofqvist et al. 2023), this review paper contributes to enhancing understanding of the role of communities in restoring landscapes across the world. This article synthesises and advances scholarship on regeneration and restoration social science, as a burgeoning field, building on and connecting to a parallel, more established research base on the role of communities in conservation and natural resource governance across the world. Building on prior scoping reviews of community engagement in ecological restoration (Fox and Cundill 2018; Reyes-Garcia 2019; Maniraho et al. 2023), we map landscape regeneration and restoration initiatives across international contexts based on four themes around community organisation, land ownership, engagement and land values.

Based on the review, we have introduced an analytical framework for community-based landscape regeneration, drawing out important themes, characteristics and trends around communities and landscape regeneration. The framework can help to guide the equitable design and development of restoration and regeneration projects. It highlights some of the opportunities and barriers around the meaningful, inclusive and sustainable involvement of communities in landscape regeneration approaches and the promises and constraints of community-based landscape regeneration.

This paper can provide impetus for further research on community-based approaches to landscape regeneration, including, for instance, in-depth examinations of specific themes and characteristics identified by the framework. There ﻿is potential for greater insights into the set of enabling conditions that facilitate community leadership on regeneration (see Chazdon et al. 2021), incorporating social, economic and political dimensions. Scholarship could build on case study research to theorise around the factors that enable and constrain community action and leadership on regeneration.

By taking stock of community-based landscape regeneration projects, programmes and practices that have been undertaken worldwide, we contribute to advancing knowledge of how communities are governing, developing, practising and implementing regeneration and restoration and the modalities, structures, livelihoods and land ownership models through which community-based restoration efforts are playing out. While building on understandings of community engagement processes in externally led projects, this review focuses on assessing regeneration and restoration initiatives led or controlled to a significant extent by communities, forms of governance which are less well understood in scholarship and policy spheres.

Moreover, this review contributes to an emerging critical research agenda on communities in the social sciences (Walker et al. 2011; Taylor Aiken et al. 2017) and political ecology spheres (Agrawal and Gibson 1999; Cooke and Kothari 2001). While dominant approaches in policy and practice typically invoke homogenous, apolitical framings of community, our review draws attention to the diversity and heterogeneity of communities engaging with restoration and regeneration practices in various ways and situated in particular social, political and geographic contexts. The power dynamics and social structures characterising the various restoration initiatives within and across rural communities limit the equity and collaborative potential of “community-based” agendas (Weng 2015), particularly given recent green investments in restoration initiatives which can further concentrate land ownership (McIntosh et al. 2023). Responding to these failures, Anguelovski and Corbera (2023) point to the strength of justice-based approaches to nature recovery which prioritises the livelihoods and needs of marginalised communities.

Drawing from our review, we propose a set of recommendations for how to realise community-based approaches to landscape regeneration in ways that do not exacerbate unequal land dynamics but instead generate more democratic and socially just approaches to ecological regeneration initiatives (see Stanley et al. 2025). These recommendations include the following:

1. Communities must be understood as diverse, heterogeneous and dynamic entities located in specific contexts.
2. Given the spectrum of forms of community engagement in practice, claims of community-based or community-led approaches to landscape regeneration must be critically assessed. It is important to capture the precise meanings of community-based approaches in place-based contexts, e.g. the role played by communities, levels of community control.
3. Aims of community participation and empowerment must go beyond the scope of projects and be situated in relation to structural barriers to participation and democratic solutions.
4. Given the dominance of market-driven approaches to the climate and biodiversity crises, the tensions between growing green investments in landscape restoration and enhancing community benefits must be managed and mitigated.
5. Further understanding is needed of how best how to support communities to sustain efforts to regenerate and restore the landscape, including beyond the typical lifecycle of a project. Community capacity-building can be supported in various ways, for example through skills training, institutional strengthening and regular sources of income.
6. Diverse knowledge forms and values must be more closely and consistently integrated into restoration and regeneration programmes.
7. Communities must be substantively included in restoration and regeneration initiatives from an early stage and throughout the different stages, for example, through co-design and collaborative planning.

## References

[CR1] Agrawal, A., and C. C. Gibson. 1999. Enchantment and disenchantment: The role of community in natural resource conservation. *World Development* 27: 629–649.

[CR107] Agrawal, A., A. Chhatre, and E.R. Gerber. 2015. Motivational crowding in sustainable development interventions. *American Political Science Review* 109: 470–487.

[CR2] Anguelovski, I., and E. Corbera. 2023. Integrating justice in nature-based solutions to avoid nature-enabled dispossession. *Ambio* 52: 45–53. 10.1007/s13280-022-01771-7 36001252 10.1007/s13280-022-01771-7 PMC9666585

[CR3] Apostolopoulou, E., A. Chatzimentor, S. Maestre-Andrés, M. Requena-i-Mora, A. Pizarro, and D. Bormpoudakis. 2021. Reviewing 15 years of research on neoliberal conservation: Towards a decolonial, interdisciplinary, intersectional and community-engaged research agenda. *Geoforum* 124: 236–256.

[CR4] Armitage, D., P. Mbatha, E. K. Muhl, W. Rice, and M. Sowman. 2020. Governance principles for community-centered conservation in the post-2020 global biodiversity framework. *Conservation Science and Practice* 2: e160.

[CR5] Ausinheiler, J. 2025. Paying to prevent deforestation is positive & not ‘nothing’. Retrieved May 2025 from: https://news.mongabay.com/2025/04/paying-to-prevent-deforestation-is-positive-not-nothing-commentary/

[CR6] Barra, M. P., and N. Jessee. 2024. Restoration as transformative reparative practice: Traditional knowledges, indigenous and black land stewardship, and solidarity. *Environment and Society* 15: 212–233.

[CR7] Baruah, M. 2017. Facipulation and elite formation: Community resource management in Southwestern Ghana. *Conservation and Society* 15: 371–383.

[CR8] Belsky, J. M. 1999. Misrepresenting communities: The politics of community-based rural ecotourism in gales point manatee, Belize. *Rural Sociology* 64: 641–666.

[CR500] Blay, D., M. Appiah, L. Damnyag, F. K. Dwomoh, O. Luukkanen, and A. Pappinen. 2008. Involving local farmers in rehabilitation of degraded tropical forests: Some lessons from Ghana. Environment, Development and Sustainability 10: 503–518.

[CR10] Bower, E., R. Harrington-Abrams, and B. Priem. 2024. Complicating “community” engagement: Reckoning with an elusive concept in climate-related planned relocation. *Global Environmental Change* 88: 102913.

[CR11] Calfucura, E. 2018. Governance, land and distribution: A discussion on the political economy of community-based conservation. *Ecological Economics* 145: 18–26.

[CR12] Calle, A. 2020. Partnering with cattle ranchers for forest landscape restoration. *Ambio* 49: 593–604. 10.1007/s13280-019-01224-8 31292911 10.1007/s13280-019-01224-8 PMC6965560

[CR105] CBD. 2022. Conference of the parties to the convention on biological diversity fifteenth meeting Part II, Montreal, Decision 15/4: The kunming-montreal Global biodiversity framework. Available at: https://www.cbd.int/doc/decisions/cop-15/cop-15-dec-04-en.pdf.

[CR13] Chazdon, R. L., P. H. Brancalion, L. Laestadius, A. Bennett-Curry, K. Buckingham, C. Kumar, J. Moll-Rocek, I. C. G. Vieira, et al. 2016. When is a forest a forest? Forest concepts and definitions in the era of forest and landscape restoration. *Ambio* 45: 538–550. 10.1007/s13280-016-0772-y 26961011 10.1007/s13280-016-0772-y PMC4980317

[CR14] Chazdon, R. L., S. J. Wilson, E. Brondizio, M. R. Guariguata, and J. Herbohn. 2021. Key challenges for governing forest and landscape restoration across different contexts. *Land Use Policy* 104: 104854.

[CR15] Cooke, B., and U. Kothari. 2001. *Participation: The new tyranny?* Zed Books.

[CR16] Coombes, B. 2007. Defending community? Indigeneity, self-determination and institutional ambivalence in the restoration of Lake Whakaki. *Geoforum* 38: 60–72.

[CR17] Creamer, E. 2015. The double-edged sword of grant funding: A study of community-led climate change initiatives in remote rural Scotland. *Local Environment* 20: 981–999.

[CR18] Curtis, P., K. Sullivan, P. Smallidge, and J. Hurst. 2021. AVID: A rapid method for assessing deer browsing of hardwood regeneration. *Forest Ecology and Management* 497: 119534.

[CR101] Denham, D. 2017. Community forest owners evaluate a decade of payments for ecosystem services in the Mexican cloud forest: The importance of attention to indigenous sovereignty in conservation. *Society & Natural Resources* 30: 1064–1079.

[CR19] Dawson, N. M., B. Coolsaet, E. J. Sterling, R. Loveridge, N. D. Gross-Camp, S. Wongbusarakum, K. K. Sangha, L. M. Scherl, et al. 2021. The role of indigenous peoples and local communities in effective and equitable conservation. *Ecology and Society* 26: 19.

[CR20] Dawson, N. M., B. Coolsaet, A. Bhardwaj, F. Booker, D. Brown, B. Lliso, J. Loos, A. Martin, et al. 2024a. Is it just conservation? A typology of Indigenous peoples’ and local communities’ roles in conserving biodiversity. *One Earth* 7: 1007–1021.

[CR21] Dawson, N. M., B. Coolsaet, A. Bhardwaj, D. Brown, B. Lliso, J. Loos, L. Mannocci, A. Martin, et al. 2024b. Reviewing the science on 50 years of conservation: Knowledge production biases and lessons for practice. *Ambio* 53: 1395–1413. 10.1007/s13280-024-02049-w 39023682 10.1007/s13280-024-02049-w PMC11383897

[CR22] Derak, M., J. Cortina, L. Taiqui, and A. Aledo. 2018. A proposed framework for participatory forest restoration in semiarid areas of North Africa. *Restoration Ecology* 26: S18–S25.

[CR100] Diver, S. 2016. Co-management as a catalyst: pathways to post-colonial forestry in the Klamath Basin, California. *Human Ecology* 44: 533–546. 10.1007/s10745-016-9851-8 PMC5099361 27881890

[CR23] Dyer, J., L. C. Stringer, A. J. Dougill, J. Leventon, M. Nshimbi, F. Chama, A. Kafwifwi, J. I. Muledi, et al. 2014. Assessing participatory practices in community-based natural resource management: Experiences in community engagement from southern Africa. *Journal of Environmental Management* 137: 137–145. 24632402 10.1016/j.jenvman.2013.11.057

[CR96] Elias, M., M., Kandel, S. Mansourian, R. Meinzen-Dick, M. Crossland, D. Joshi, J. Kariuki, L. C. Lee. et al. 2022. Ten people‐centered rules for socially sustainable ecosystem restoration restoration. *Ecology* 30. 10.1111/rec.13574

[CR24] Elliott, S., S. Chairuangsri, C. Kuaraksa, S. Sangkum, K. Sinhaseni, D. Shannon, P. Nippanon, and B. Manohan. 2019. Collaboration and conflict—developing forest restoration techniques for northern Thailand’s upper watersheds whilst meeting the needs of science and communities. *Forests* 10: 732.

[CR25] Erbaugh, J. T., N. Pradhan, J. Adams, J. A. Oldekop, A. Agrawal, D. Brockington, R. Pritchard, and A. Chhatre. 2020. Global forest restoration and the importance of prioritizing local communities. *Nature Ecology and Evolution* 4: 1472–1476. 32839542 10.1038/s41559-020-01282-2

[CR26] Ewane, E. B. 2024. Understanding community participation in tree planting and management in deforested areas in Cameroon’s Western Highlands. *Environmental Management* 73: 274–291. 37882834 10.1007/s00267-023-01902-0

[CR27] Fabricius, C., and T. Pereira. 2015. Community biodiversity inventories as entry points for local ecosystem stewardship in a South African communal area. *Society and Natural Resources* 28: 1030–1042.

[CR99] Fairhead, J., M. Leach, and I. Scoones. 2012. Green grabbing: a new appropriation of nature?. *Journal of Peasant Studies* 39: 237–261.

[CR28] Fischer, H. W., A. Chhatre, A. Duddu, N. Pradhan, and A. Agrawal. 2023. Community forest governance and synergies among carbon, biodiversity and livelihoods. *Nature Climate Change* 13: 1340–1347.

[CR102] Flood, K., M. Mahon, and J. McDonagh. 2022. Everyday resilience: Rural communities as agents of change in peatland social-ecological systems. *Journal of Rural Studies* 96: 316–331.

[CR29] Fox, H., and G. Cundill. 2018. Towards increased community-engaged ecological restoration: A review of current practice and future directions. *Ecological Restoration* 36: 208–218.

[CR30] Fox, C. A., N. J. Reo, D. A. Turner, J. Cook, F. Dituri, B. Fessell, J. Jenkins, A. Johnson, et al. 2017. “The river is us; the river is in our veins”: Re-defining river restoration in three Indigenous communities. *Sustainability Science* 12: 521–533.

[CR104] Gabrys, J., M. Westerlaken, and Y.A. Fatimah. 2025. Actually existing smart forests: A proposal for pluralizing eco-technical worlds. *Environment and Planning F* 4: 210–232.

[CR31] Gabrys, J., M. Westerlaken, D. Urzedo, M. Ritts, and T. Simlai. 2022. Reworking the political in digital forests: The cosmopolitics of socio-technical worlds. *Progress in Environmental Geography* 1: 58–83.

[CR33] Gann, G. D., T. McDonald, B. Walder, J. Aronson, C. R. Nelson, J. Jonson, J. G. Hallett, C. Eisenberg, et al. 2019. International principles and standards for the practice of ecological restoration. *Restoration Ecology* 27: S1–S46.

[CR34] Gómez-Ruiz, P. A., R. A. Betancourth-Buitrago, M. Arteaga-Cote, J. P. Carbajal-Borges, C. Teutli-Hernández, and S. Laffon-Leal. 2022. Fostering a participatory process for ecological restoration of mangroves in Pantanos de Centla Biosphere Reserve (Tabasco, Mexico). *Ecosystems and People* 18: 112–118.

[CR35] Gregorio, N., J. Herbohn, R. Tripoli, and A. Pasa. 2020. A local initiative to achieve global forest and landscape restoration challenge—Lessons learned from a community-based forest restoration project in Biliran province, Philippines. *Forests* 11: 475.

[CR36] Guibrunet, L., P. R. W. Gerritsen, J. A. Sierra-Huelsz, A. C. Flores-Díaz, E. García-Frapolli, E. García-Serrano, U. Pascual, and P. Balvanera. 2021. Beyond participation: How to achieve the recognition of local communities’ value-systems in conservation? Some insights from Mexico. *People and Nature* 3: 528–541.

[CR103] Hesley, D., D. Burdeno, C. Drury, S. Schopmeyer, and D. Lirman. 2017. Citizen science benefits coral reef restoration activities. *Journal for Nature Conservation* 40: 94–99.

[CR37] Hegwood, M., R. E. Langendorf, and M. G. Burgess. 2022. Why win–wins are rare in complex environmental management. *Nature Sustainability* 5: 674–680.

[CR38] Hemmerling, S. A., C. DeMyers, J. Parfait, E. Piñero, M. M. Baustian, M. Bregman, D. Di Leonardo, C. Esposito, et al. 2023. A community-informed transdisciplinary approach to coastal restoration planning: Maximizing the social and ecological co-benefits of wetland creation in Port Fourchon, Louisiana, USA. *Frontiers in Environmental Science* 11: 1105671.

[CR39] Hinojosa-Huerta, O., M. Briggs, Y. Carrillo-Guerroro, E.P. Glenn, M. Lara-Flores, and M. Román-Rodríguez. 2005. Community-based restoration of desert wetlands: the case of the Colorado River Delta. In *Bird Conservation Implementation and Integration in the Americas: Proceedings of the Third International Partners in Flight Conference.* Eds. R.C. John, and T.D. Rich, 637–645. Albany, CA: U.S. Dept. of Agriculture, Forest Service, Pacific Southwest Research Station.

[CR40] Huber, J. M., J. Newig, and J. Loos. 2023. Participation in protected area governance: A systematic case survey of the evidence on ecological and social outcomes. *Journal of Environmental Management* 336: 117593. 36947956 10.1016/j.jenvman.2023.117593

[CR41] Kamelamela, K. L., H. K. Springer, R. K. U. Keakealani, M. U. Ching, T. Ticktin, R. D. Ohara, E. W. Parsons, E. D. Adkins, et al. 2022. Kōkua aku, Kōkua mai: An Indigenous consensus-driven and place-based approach to community led dryland restoration and stewardship. *Forest Ecology and Management* 506: 119949.

[CR42] Kandel, M., G. Agaba, R. S. Alare, T. Addoah, and K. Schreckenberg. 2021. Assessing social equity in farmer-managed natural regeneration (fmnr) interventions: Findings from Ghana. *Ecological Restoration* 39: 64–76.

[CR43] Kandel, M., D. Anghileri, R. S. Alare, P. N. Lovett, G. Agaba, T. Addoah, and K. Schreckenberg. 2022. Farmers’ perspectives and context are key for the success and sustainability of farmer-managed natural regeneration (FMNR) in northeastern Ghana. *World Development* 158: 106014.

[CR44] Kenis, A., and E. Mathijs. 2014. (De) politicising the local: The case of the transition towns movement in Flanders (Belgium. *Journal of Rural Studies* 34: 172–183.

[CR45] Kothari, A., P. Camill, and J. Brown. 2013. Conservation as if people also mattered: Policy and practice of community-based conservation. *Conservation and Society* 11: 1–15.

[CR46] Lasco, R. D., R. S. Evangelista, and F. B. Pulhin. 2010. Potential of community-based forest management to mitigate climate change in the Philippines. *Small-Scale Forestry* 9: 429–443.

[CR47] Latulippe, N., and N. Klenk. 2020. Making room and moving over: Knowledge co-production, indigenous knowledge sovereignty and the politics of global environmental change decision-making. *Current Opinion in Environmental Sustainability* 42: 7–14.

[CR48] Löfqvist, S., F. Kleinschroth, A. Bey, A. De Bremond, R. DeFries, J. Dong, F. Fleischman, S. Lele, et al. 2023. How social considerations improve the equity and effectiveness of ecosystem restoration. *BioScience* 73: 134–148. 36896142 10.1093/biosci/biac099 PMC9991587

[CR49] Loveridge, R., A. R. Marshall, M. Pfeifer, S. Rushton, P. P. Nnyiti, L. Fredy, and S. M. Sallu. 2023. Pathways to win-wins or trade-offs? How certified community forests impact forest restoration and human wellbeing. *Philosophical Transactions of the Royal Society B* 378: 20210080. 10.1098/rstb.2021.0080 PMC9661953 36373927

[CR50] Lund, J. F. 2015. Paradoxes of participation: The logic of professionalization in participatory forestry. *Forest Policy and Economics* 60: 1–6.

[CR51] Lupp, G., A. Zingraff-Hamed, J. J. Huang, A. Oen, and S. Pauleit. 2020. Living labs—a concept for co-designing nature-based solutions. *Sustainability* 13: 188.

[CR52] Lyver, P. O. B., A. Akins, H. Phipps, V. Kahui, D. R. Towns, and H. Moller. 2016. Key biocultural values to guide restoration action and planning in New Zealand. *Restoration Ecology* 24: 314–323.

[CR53] M'Sit No'kmaq, A., K. F. Marshall, J. Beazley, S. Hum, A. Joudry, S. Papadopoulos, J. Pictou, L. Y. Rabesca, et al. 2021. “Awakening the sleeping giant”: re-Indigenization principles for transforming biodiversity conservation in Canada and beyond. *Facets* 6: 839–869.

[CR54] Mabele, M. B., J. E. Krauss, and W. Kiwango. 2022. Going back to the roots: Ubuntu: And just conservation in Southern Africa. *Conservation and Society* 20: 92–102.

[CR55] Maniraho, L., M. Frietsch, S. Sieber, and K. Löhr. 2023. A framework for drivers fostering social-ecological restoration within forest landscape based on people’s participation. A systematic literature review. *Discover Sustainability* 4: 26.

[CR56] Mansourian, S., L. Aquino, T. K. Erdmann, and F. Pereira. 2014. A comparison of governance challenges in forest restoration in Paraguay’s privately-owned forests and Madagascar’s co-managed state forests. *Forests* 5: 763–783.

[CR57] Markantoni, M., and M. Woolvin. 2015. The role of rural communities in the transition to a low-carbon Scotland: A review. *Local Environment* 20: 202–219.

[CR58] Martin, A., A. Fischer, and R. McMorran. 2023. Who decides? The governance of rewilding in Scotland ‘between the cracks’: Community participation, public engagement, and partnerships. *Journal of Rural Studies* 98: 80–91.

[CR109] Mawutor, S. M. and R. Hajjar. 2022. Examining the powers decentralized to community resource management areas in Ghana. *Land Use Policy* 119.

[CR59] McElwee, P. 2011. Who should manage the land? Common property and community responses in Vietnam’s shifting uplands. In *Upland transformation in Vietnam*, ed. T. Sikor, 75–91. Singapore: National University of Singapore Press.

[CR60] McIntosh, A. 2023. The cheviot, the stag and the black, black carbon: Natural capital, the private finance investment pilot and Scotland’s land reform. Report, Community Land Scotland, UK. Retrieved from: https://www.communitylandscotland.org.uk/wp-content/uploads/2023/05/2023-CLS-Full-Cheviot-Carbon-Discussion-McIntosh.docx.pdf

[CR61] Mehta, L., S. Srivastava, H. N. Adam, B. S. Alankar, U. Ghosh, and V. V. Kumar. 2019. Climate change and uncertainty from ‘above’ and ‘below’: Perspectives from India. *Regional Environmental Change* 19:1533–1547.

[CR62] Murphy, S. P., S. Cannon, and L. Walsh. 2022. Just transition frames: Recognition, representation, and distribution in Irish beef farming. *Journal of Rural Studies* 94: 150–160.

[CR63] Mustonen, T., A. Scherer, and J. Kelleher. 2022. We belong to the land: Review of two northern rewilding sites as a vehicle for equity in conservation. *Humanities and Social Sciences Communications* 9: 1–9.

[CR64] Mutillod, C., E. Buisson, G. Mahy, R. Jaunatre, J. M. Bullock, L. Tatin, and T. Dutoit. 2024. Ecological restoration and rewilding: Two approaches with complementary goals? *Biological Reviews* 99: 820–836. 38346335 10.1111/brv.13046

[CR65] Oldekop, J. A., G. Holmes, W. E. Harris, and K. L. Evans. 2016. A global assessment of the social and conservation outcomes of protected areas. *Conservation Biology* 30: 133–141. 26096222 10.1111/cobi.12568

[CR95] Olukoye, G. A., and J. I. Kinyamario. 2009. Community participation in the rehabilitation of a sand dune environment in Kenya. *Land Degradation & Development* 20: 397–409.

[CR98] Orlove, B., P. Sherpa, N. Dawson, I. Adelekan, W. Alangui, R. Carmona, D. Coen, M.K. Nelson, V. Reyes-García, J. Rubis, and G. Sanago. 2023. Placing diverse knowledge systems at the core of transformative climate research. *Ambio* 52: 1431–1447. 10.1007/s13280-023-01857-w PMC10406791 37103778

[CR97] Osborne, T., S. Brock, R. Chazdon, S. Chomba, E. Garen, V. Gutierrez, R. Lave, M. Lefevre, and J. Sundberg. 2021. The political ecology playbook for ecosystem restoration: Principles for effective, equitable, and transformative landscapes. *Global Environmental Change* 70.

[CR66] Pascual, U., W. M. Adams, S. Díaz, S. Lele, G. M. Mace, and E. Turnhout. 2021. Biodiversity and the challenge of pluralism. *Nature Sustainability* 4: 567–572.

[CR67] Pasgaard, M., C.A. Breed, M. Heins, L. Knudsen, P. Brom, Schmidt, A. and Engemann, K., 2023. Citizen science beyond science: A collaborative approach for transformative sustainable development.

[CR68] Peterson, R. B., R. A. Kapiyo, E. M. Campbell, and P. O. Nyabua. 2018. Gully rehabilitation trusts: Fighting soil erosion through community participation in western Kenya. *Journal of Rural Studies* 58: 67–81.

[CR69] Poffenberger, M. 2015. Restoring and conserving Khasi forests: A community-based REDD strategy from northeast India. *Forests* 6: 4477–4494.

[CR70] Ptak, T., A. Nagel, S. M. Radil, and D. Phayre. 2018. Rethinking community: Analyzing the landscape of community solar through the community-place nexus. *The Electricity Journal* 31: 46–51.

[CR71] Rao, K. S., R. L. Semwal, S. Ghoshal, R. K. Maikhuri, S. Nautiyal, and K. G. Saxena. 2022. Participatory active restoration of communal forests in temperate Himalaya, India. *Restoration Ecology* 30: e13486.

[CR72] Reyes-García, V., Á. Fernández-Llamazares, P. McElwee, Z. Molnár, K. Öllerer, S. J. Wilson, and E. S. Brondizio. 2019. The contributions of indigenous peoples and local communities to ecological restoration. *Restoration Ecology* 27: 3–8.

[CR73] Ribot, J. C., J. F. Lund, and T. Treue. 2010. Democratic decentralization in sub-Saharan Africa: Its contribution to forest management, livelihoods, and enfranchisement. *Environmental Conservation* 37: 35–44.

[CR74] Sanches, R. A., C. R. T. Futemma, and H. Q. Alves. 2021. Indigenous territories and governance of forest restoration in the Xingu River (Brazil). *Land Use Policy* 104: 104755.

[CR75] Shaban, K. S., J. O. Okumu, O. Paul, and O. Joseph. 2016. Assessing community-based organizations’ influence on trees and grass planting for forest, soil and water management around Mt. Elgon National Park in Uganda. *Forests, Trees and Livelihoods* 25: 161–172.

[CR76] Sharma, K., J. Hollingdale, G. Walters, M. J. Metzger, and J. Ghazoul. 2023. In danger of co-option: Examining how austerity and central control shape community woodlands in Scotland. *Geoforum* 142: 103771.

[CR77] Stanley, T., M. Hirons, J. Turnbull, J. Lorimer, E. M. Kumeh, C. Hafferty, L. M. Anderson, and C. L. McDermott. 2025. Just nature recovery: A framework for centring multispecies and multi-dimensional justice in land management. *Environmental Science and Policy* 164: 103992.

[CR78] Stanley, T. 2024. Technical wildness: Reforestation, rewilding, and the environmental performativity of forest measurement in the Scottish Highlands. PhD Thesis. Oxford, United Kingdom: University of Oxford.

[CR79] Stenseke, M. 2009. Local participation in cultural landscape maintenance: Lessons from Sweden. *Land Use Policy* 26: 214–223.

[CR80] Sullivan, J. J., and L. E. Molles. 2016. Biodiversity monitoring by community-based restoration groups in New Zealand. *Ecological Management and Restoration* 17: 210–217.

[CR81] Sze, J. S., L. R. Carrasco, D. Childs, and D. P. Edwards. 2022. Reduced deforestation and degradation in Indigenous Lands pan-tropically. *Nature Sustainability* 5: 123–130.

[CR83] Taylor Aiken, G., L. Middlemiss, S. Sallu, and R. Hauxwell-Baldwin. 2017. Researching climate change and community in neoliberal contexts: An emerging critical approach. *Wiley Interdisciplinary Reviews: Climate Change* 8: e463.

[CR106] The Langholm Initiative. 2025. Creating the Tarras valley nature reserve. Retrieved 15 May 2025 from: https://www.tarrasvalleynaturereserve.org/wp-content/uploads/2025/02/TVN008_Report25_Spreads-5.pdf

[CR84] Tidball, K. G., S. Metcalf, M. Bain, and T. Elmqvist. 2018. Community-led reforestation: Cultivating the potential of virtuous cycles to confer resilience in disaster disrupted social–ecological systems. *Sustainability Science* 13: 797–813.

[CR108] Turnhout, E., S. Van Bommel. and N. Aarts. 2010. How participation creates citizens: participatory governance as performative practice. *Ecology and Society* 15.

[CR85] Urzedo, D., M. Westerlaken, and J. Gabrys. 2022a. Digitalizing forest landscape restoration: A social and political analysis of emerging technological practices. *Environmental Politics* 32: 485–510. 37207120 10.1080/09644016.2022.2091417 PMC10191160

[CR86] Urzedo, D., S. Pedrini, D. L. Vieira, A. B. Sampaio, B. D. Souza, E. M. Campos-Filho, F. C. Pina-Rodrigues, I. B. Schmidt, et al. 2022b. Indigenous and local communities can boost seed supply in the UN decade on ecosystem restoration. *Ambio* 51: 557. 10.1007/s13280-021-01593-z 34231132 10.1007/s13280-021-01593-z PMC8259771

[CR87] Vasile, M. 2018. The vulnerable bison: Practices and meanings of rewilding in the Romanian Carpathians. *Conservation and Society* 16: 217–231.

[CR88] Walker, G. 2011. The role for ‘community’ in carbon governance. *Wiley Interdisciplinary Reviews: Climate Change* 2: 777–782.

[CR89] Weng, Y. C. 2015. Contrasting visions of science in ecological restoration: Expert-lay dynamics between professional practitioners and volunteers. *Geoforum* 65: 134–145.

[CR90] Weston, P., R. Hong, C. Kaboré, and C. A. Kull. 2015. Farmer-managed natural regeneration enhances rural livelihoods in dryland West Africa. *Environmental Management* 55: 1402–1417. 25835946 10.1007/s00267-015-0469-1

[CR91] Wilson, S. J., and O. T. Coomes. 2019. ‘Crisis restoration’ in post-frontier tropical environments: Replanting cloud forests in the Ecuadorian Andes. *Journal of Rural Studies* 67: 152–165.

[CR92] Wilson, S. J., and J. M. Rhemtulla. 2016. Acceleration and novelty: Community restoration speeds recovery and transforms species composition in Andean cloud forest. *Ecological Applications* 26: 203–218. 27039520 10.1890/14-2129

[CR93] Wiset, K., N. Gregorio, R. Fisher, E. Mangaoang, and J. Herbohn. 2023. Assessing the effectiveness of the engagement of local people in restoring degraded forest landscapes in Leyte and Biliran Provinces, the Philippines. *Environmental Science AND Policy* 148: 103545.

[CR94] Zhang, Y., P. West, L. Thakholi, K. Suryawanshi, M. Supuma, D. Straub, S. S. Sithole, R. Sharma, et al. 2023. Governance and conservation effectiveness in protected areas and indigenous and locally managed areas. *Annual Review of Environment and Resources* 48: 559–588.

